# Multilayered safety framework for living diagnostics in the colon

**DOI:** 10.3389/fsysb.2023.1240040

**Published:** 2023-09-22

**Authors:** Sonia Mecacci, Lucía Torregrosa-Barragán, Enrique Asin-Garcia, Robert W. Smith

**Affiliations:** ^1^ Laboratory of Systems and Synthetic Biology, Wageningen University & Research, Wageningen, Netherlands; ^2^ Bioprocess Engineering, Wageningen University & Research, Wageningen, Netherlands

**Keywords:** safe-by-design, diagnostic tool, inducible kill switches, colorectal cancer, genetic circuits, biosafety, biocontainment, auxotrophy

## Abstract

**Introduction:** Colorectal cancer is the second most deadly cancer worldwide. Current screening methods have low detection rates and frequently provide false positive results, leading to missed diagnoses or unnecessary colonoscopies. To tackle this issue, the Wageningen UR iGEM team from 2022 developed “Colourectal”, a living diagnostic tool for colorectal cancer. Following a synthetic biology approach, the project used an engineered *Escherichia coli* Nissle 1917 strain capable of binding to tumour cells that detects two distinct cancer biomarkers, and secretes a coloured protein observable in stool. Due to the utilization of genetically modified bacteria *in vivo*, precautionary biosafety measures were included within a three level safe-by-design strategy.

**Results:** The first genetic safeguard ensured confinement of the living diagnostic to the colon environment by implementing auxotrophy to mucin that is abundant in the colon lining. For this, a synthetic chimeric receptor was generated to ensure expression of essential genes in the presence of mucin. The second strategy limited the viability of the engineered bacteria to the human body, preventing proliferation in open environments. The use of a temperature sensitive kill switch induced bacterial cell death at temperatures below 37°C. The third biocontainment strategy was installed as an emergency kill switch to stop the Colourectal test at any point. By inducing a highly genotoxic response through CRISPR-Cas-mediated DNA degradation, cell death of *E. coli* Nissle is triggered.

**Discussion:** While the use of engineered microorganisms in human applications is not yet a reality, the safety considerations of our multi-layered strategy provide a framework for the development of future living diagnostic tools.

## Introduction

Colorectal cancer (CRC) is the third most common type of cancer and the second leading cause of cancer deaths in the world (Sung et al., 2021; Siegel et al., 2022). When detected in early stages, the 5-year survival rate is 91%. However, when detected in late stages of the disease, this rate dramatically drops to 14%. This makes early detection of CRC a critical need to prevent unnecessary deaths. For this reason, in the Netherlands, CRC screenings are carried out every 2 years for those aged 55 to 75 using the Faecal Immunochemical Test (FIT) (RIVM, www.rivm.nl/documenten/monitoring-colorectal-cancer-screening-programme-2020). If the FIT reports traces of blood in the stool, indicative of CRC, the user is invited to undergo a colonoscopy to verify whether they have cancer. However, in 2021, only 31.5% of unfavourable FITs corresponded to the presence of CRC or advanced adenoma (AAD) when verified by colonoscopy. This is evidence of the high false positive rate associated with FIT, which places a high burden on the healthcare system as well as on the mental health of users.

**FIGURE 1:**
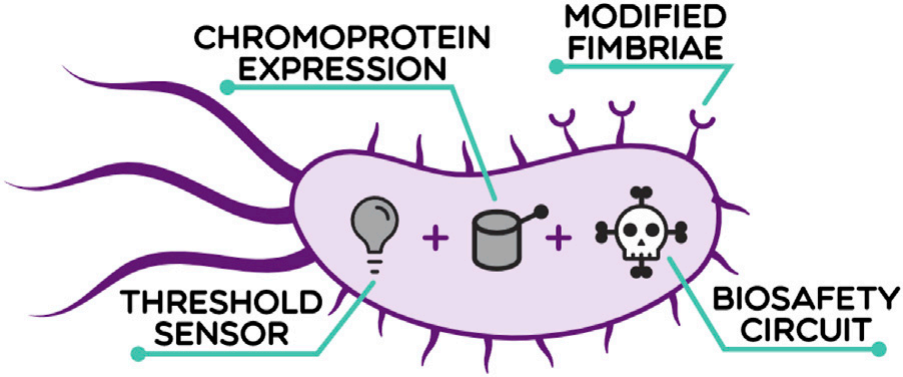
The living diagnostic *E. coli* Nissle 1917 (EcN), referred to as Colourectal, is engineered to sense and report colorectal cancer. Colourectal is based on four pillars: interaction, detection, signalling and biosafety. When the living diagnostic accesses the colon, it first migrates and binds to cancer cells (interaction) when a tumour is present. If the two CRC biomarkers are present, high levels of lactate and the presence of protein matrix metalloproteinase 9, are present then activation of the test occurs within the engineered bacteria (detection). Only if both biomarkers are present, a coloured protein is secreted as a response. This colour change is then visible in stool (signalling). Finally, a biocontainment plan has been implemented on three levels to diminish any possible risk associated with the living diagnostic (biosafety, this paper).

The 2022 Wageningen University and Research iGEM team aimed to address the need for an early, more reliable diagnostic method for CRC and worked on the development of a living diagnostic tool named Colourectal (Wageningen_UR 2022; https://2022.igem.wiki/wageningen-ur/). The reason why Colourectal is referred to as a “living diagnostic” is due to its mechanism, which is based on the *in vivo* action of a microorganism that would be ingested by the user, travel through the colon environment in search of CRC, and report its presence by secreting a blue chromoprotein that would be visible in the stool (Figure 1). Additionally, three layers of biosafety, which are the focus of this work, were investigated with future applications of Colourectal in mind. The microorganism chosen to perform this function was the probiotic strain *Escherichia coli* Nissle 1917 (EcN). EcN is safe for human consumption and has been commercialised in multiple countries due to its probiotic properties (Sonnenborn, 2016). Furthermore, it is also one of the most widely engineered probiotic strains for use in the diagnosis or treatment of various diseases, such as obesity or cancer (Rottinghaus et al., 2022). In addition, due to its strong genetic resemblance to the model *E. coli* strains, there is an extensive toolbox available for its genetic manipulation (Ou et al., 2016).

Advances in genetic engineering technologies have allowed non-model organisms and strains to be modified, making microbiome microorganisms accessible chassis (Mimee et al., 2015; Zuo et al., 2020). Consequently, new applications of engineered probiotics for diagnostic and (bio)therapeutic purposes have gained popularity (Riglar and Silver, 2018; Pedrolli et al., 2019; Charbonneau et al., 2020). Examples range from engineering organisms to deliver site-specific therapeutic molecules (Hwang et al., 2017; Plamer et al., 2018; He et al., 2019), to strains used as prophylactic vaccines (Foligne et al., 2007; Lagenaur et al., 2011), or to sense and diagnose diseases in the gut (Daeffler et al., 2017; Riglar et al., 2017).

The increased use of engineered organisms generates important questions about safety. Possible risks are related to the release of synthetic genetic material, which, in turn, could affect human, animal, plant and ecosystem health (Beeckman and Rüdelsheim, 2020; Pantoja Angles et al., 2022). To mitigate these concerns, a safe-by-design approach should be followed to tackle possible threats before they might occur (Asin-Garcia et al., 2020). Up to now, explored containment strategies in biotherapeutics include auxotrophic approaches, whereby system survival is dependent on an environmental cue (Kunjapur et al., 2021), and kill switches (Piraner et al., 2017; Riglar et al., 2017; Rottinghaus et al., 2022). Combined strategies have also been studied to strengthen biocontainment, tackling evolutionary instability and organisms that may escape the effects of a single strategy (Gallagher et al., 2015; Stirling et al., 2017). Moreover, a multi-layered system is envisioned to achieve three main properties: low escape frequency, robustness, and modularity (Gallagher et al., 2015). In microbiome engineering, a similar multi-layered approach has been explored for *Saccharomyces boulaurdii*, that is studied for its ability to synthetise and fold relevant therapeutic proteins (Hedin et al., 2023). In addition to the choice of a safe chassis, a combination of auxotrophy and temperature-sensitive biocontainment was applied to safeguard the release of *S. boulaurdii* to the environment (Hedin et al., 2023). In line with these approaches, we also envisioned a multi-layered biocontainment strategy for our system. To ensure safety on all levels we used three different approaches: an auxotrophy to confine the living diagnostic to the colon, a temperature-sensitive kill switch to avoid environmental escape, and an inducible kill switch to allow to control the living diagnostic at any time.

First, an auxotrophy was used in our system to prevent the spread of the living diagnostic from the colon to other parts of the body. The migration and colonization of other body tissues could cause conditions such as bacteraemia, the presence of bacteria in the bloodstream, and in the worst cases, sepsis, an infection with a systemic inflammatory response (Evans, 2018; Smith and Nehring, 2023). Auxotrophies have been implemented previously in other engineered biotherapeutics for *in vivo* and environmental biocontainment (Isabella et al., 2018; Kurtz et al., 2019). We hypothesized that building an auxotrophic system that makes the engineered strain dependent to the colon environment will avoid the spread to other body parts. Our living diagnostic should be able to sense, respond, and survive only upon the presence of a specific stimulus. Mucins, which are large, heavily *O*-glycosylated glycoproteins, are the main protein component of mucus and are responsible for mucus’ viscous properties (McGuckin et al., 2011). They are continuously produced by the cells lining the gastrointestinal tract and are, therefore, always present in the colon (Sicard et al., 2017). Thus, they are an ideal candidate for the envisioned auxotrophy. As such, the system would only survive in the presence of mucus in the gastrointestinal tract and would not be able to survive in other areas of the body.

To prevent the escape of our organism in the environment, we used multiple kill switches. Kill switches respond to environmental inputs such that the microbes survive specifically in permissive environments. Upon exit of these permissive conditions, the lethal components become active, leading to cell death (Rottinghaus et al., 2022). Such genetic circuits can be built with a variety of inputs, ranging from physical inputs such as temperature (Piraner et al., 2017; Stirling et al., 2017) to chemical inputs such as inducer molecules (Knudsen and Karlström, 1991; Caliando and Voigt, 2015; Rottinghaus et al., 2022). The multi-layered biosafety strategy that we envisioned for Colourectal required that the two kill switches are activated by different triggers and with different killing mechanisms. These triggers would act in addition to the mucin auxotrophy discussed above. First, to prevent the proliferation of the genetically modified organisms outside the body we envisioned a switch in temperature as the perfect trigger to activate cell death. Ideally, the system would allow cell survival only at the permissive condition of 37 °C, the human body temperature, whereas the kill switch would become activated when the temperature drops, as would occur when Colourectal leaves the body. Temperature-sensitive kill switches have been already employed for containment of living therapeutics and diagnostics (Piraner et al., 2017; Stirling et al., 2017). In our envisioned application, the first kill switch is activated by a physical stimulus and uses a toxin as a killing mechanism, the second is triggered by a chemical inducer and initiates cell death by targeting the genome with CRISPR-Cas.

In tandem with this second instance, and on top of safety precautions, we also wanted to be able to remove the *in vivo* diagnostic from the colon system on demand. We envisioned this would be needed when the diagnosis has been completed, or in the event of an unexpected adverse effect. Many kill switches have been developed for living therapeutics in which the presence of a chemical inducer initiates cell death (Pedrolli et al., 2019; Rottinghaus et al., 2022). A suitable inducible expression system for Colourectal must be activated by a molecule that is not harmful to human health at the concentrations required for induction, that is not already present in the human gut environment, and that is not part of the human diet. In addition, it must remain inactive under permissive conditions, while generating a strong response in the presence of the inducer. Recent research has shown that the salicylate derivative, acetylsalicylic acid (ASA, commonly known as aspirin), can be used to induce genetic circuits (de Lorenzo et al., 1993; Medina et al., 2011; Monteiro et al., 2019). Therefore, we believed that ASA could be a promising inducer for our third kill switch given its use in other medical treatments. Additionally, other chemical inducers such as sugars (e.g., arabinose and rhamnose) and benzoates have been widely used to regulate the expression of genetic circuits in *E. coli* (Guzman et al., 1995; Loessner et al., 2009; Gawin et al., 2017), and could be good alternatives for our application.

To trigger cell death, the inducer molecule activates a CRISPR-Cas system. These systems have been widely used for the development of kill switches that precisely and specifically target genetic elements (Caliando and Voigt, 2015; Csörgő et al., 2020; Rottinghaus et al., 2022). Such strategies make it possible to effectively eliminate the desired bacteria while leaving the rest of the microbial community unharmed. Class I, type I CRISPR-Cas systems are comprised of a variable number of Cas proteins that, together with crRNA, form an effector module called Cascade. Cascade recruits the Cas3 subunit, common to all type I systems, to cleave target DNA that must be complementary to the spacer sequence contained within the crRNA and located next to the PAM (Protospacer Adjacent Motif) (Nimkar and Anand, 2020). An inducible type I-E CRISPR-Cas-based kill switch was developed in *E. coli* with the ability to degrade the genome in a fast and targeted way, reducing the number of viable cells by approximately 99.99% (Caliando and Voigt, 2015). Additionally, this circuit was demonstrated to be non-toxic for the cell population over a period of months when not induced. As well as the type I-E CRISPR-Cas system, type I-C CRISPR-Cas has been highlighted as an option for genomic degradation kill switches (Csörgő et al., 2020). Although the authors applied the system for gene editing, large deletions (10s–100s of kilobases) on both sides of the crRNA target site were also reported when targeting the genome of *E. coli* which we hypothesised would cause a highly genotoxic effect.

In this work, we constructed a multi-layered biocontainment system in EcN based on a mucin auxotrophy, a temperature-sensitive kill switch and a rhamnose-inducible CRISPR-Cas-based kill switch. We designed an auxotrophy on mucin to ensure the confinement to the colon environment. This was achieved with the construction of the chimeric receptor Dismed2:EnvZ that responds to mucin. We then tested the temperature-sensitive kill switch based on pcspA and a toxin/antitoxin system that showed 100% efficiency. Finally, we developed a test-terminator kill switch to allow removal of the living diagnostic from the colon at any desired time. The circuit was inducible by rhamnose and based on the type I-C CRISPR-Cas mechanism, targeting multiple loci in the genome of EcN. We propose a robust biosafety and biocontainment strategy that can be implemented in the design of future engineered microorganisms to be used in the human body for therapeutic or diagnostic purposes.

## Materials and methods

### Strains, plasmids and bacterial growth conditions

The bacterial strains used in this study along with their respective characteristics can be found in Supplementary Table S1. *E. coli* DH5α was made chemically competent (Green and Rogers, 2013) for cloning purposes. EcN and *E. coli* JW3367-3 ∆*envZ* were made chemically competent (Green and Rogers, 2013) or electrochemically competent for growth assays, fluorescence assays, and temperature assays. Unless otherwise stated, all the strains were cultured on LB medium at 37 °C. Antibiotics were added when required at the following concentrations: kanamycin, 50 mg/mL; chloramphenicol, 31 mg/mL. Growth experiments and fluorescence assays were performed in M9 minimal medium (1.63 g/L NaH_2_PO_4_, 3.88 g/L K_2_HPO_4_, 2 g/L (NH_4_)_2_SO_4_, 10 mg/L EDTA, 100 mg/L MgCl_2_·6H_2_O, 2 mg/L ZnSO_4_·7H_2_O, 1 mg/L CaCl_2_·2H_2_O, 5 mg/L FeSO_4_·7H_2_O, 0.2 mg/L Na_2_MoO_4_·2H_2_O, 0.2 mg/L CuSO_4_·5H_2_O, 0.4 mg/L CoCl_2_·6H_2_O, and 1 mg/L MnCl_2_·2H_2_O) supplemented with 50 mM of glucose. For the different experiments, media was supplemented with either 0.1% (v/v), 1% (v/v), 5% (v/v) and 10% (v/v) Hog gastric mucin (Sigma); 100 μM, 300 μM, 500 μM, 1 mM, 3 mM, 5 mM, and 10 mM L-rhamnose inducer; or 2 mM of ASA (aspirin), 3MB (benzoate), or L-arabinose.

### Dismed2:EnvZ chimeric receptor domain analysis

Domain analysis of the *P. aeruginosa* PA01 RetS protein was performed with SMART (Letunic et al., 2021) and DeepTMHMM (Hallgren et al., 2022) to ascertain the amino acid sequence of Dismed2. The retrieved nucleotide sequence of *E. coli* EnvZ from a previous iGEM project (Wageningen_UR 2019, https://2019.igem.org/Team:Wageningen_UR) was aligned with BLASTn to the *Escherichia coli* Nissle 1917 (taxid:316435) genome (Zhang et al., 2000). The EcN sequence was checked by the bioinformatic analysis tools SMART and DeepTMHMM. The amino acid sequence of the chimeric receptor Dismed2:EnvZ was then examined with DeepTMHMM and PSORTb v3.0 (Yu et al., 2010) to predict the location of the chimeric protein within the cell. The OmpC promoter region retrieved from *E. coli* was also checked with BLASTn for similarity with EcN. BLAST, SMART, Psortb and DeepTMHMM were used using the default settings.

### Plasmid cloning

To construct the plasmids used in this study (Supplementary Table S2), the following workflow was performed. PCR amplification of all the genetic parts was done with specific primers (Supplementary Table S3) using the NEB Q5 High-Fidelity DNA polymerase. PCR products were run in a 1% w/v agarose gel electrophoresis. DNA bands of the correct size were excised from the gel and purified using Nucleospin Gel and PCR clean-up kit (Machery-Nagel) according to the manufacturer instructions. Assembly was performed using the Golden Gate-based SEVABrick Assembly method (Damalas et al., 2020) and transformed into chemically competent *E. coli* DH5α cells to amplify the assembled plasmid. Successful transformation was verified by colony PCR with Phire Hot Start II polymerase. Plasmids from verified colony PCR samples were then isolated from liquid cultures using the GeneJET plasmid Miniprep kit (Thermo Fisher), following the manufacturer’s instructions. Plasmid cargo sequences were verified by Sanger sequencing from Macrogen (MACROGEN Inc. DNA Sequencing Service; Amsterdam, Netherlands) and, when necessary, whole plasmids sequences were verified by Oxford Nanopore plasmid sequencing from Plasmidsaurus (SNPsaurus, Eugene, US).

To construct the Dismed2:EnvZ chimeric receptor, first, the Dismed2 sequence was amplified from the genome of *P. aeruginosa* with primers C1+C2, and *envZ* from the genome of EcN with C3+C4 (Supplementary Table S2). Then, they were assembled together in a pSB1C3 backbone. The Dismed2:EnvZ coding sequence was then amplified with primers U3+U4. The Dismed2:EnvZ was then cloned into the pSEVAb22 plasmid under the control of the constitutive BBa_J23100 Anderson promoter. Construction of pSB1C3_pOmpC-GFP was done after amplification of the OmpC promoter from the genome of EcN with primers C5+C6, and *gfp* from the in-house plasmid pSEVA238 D.M gfp with C7+C8.

The plasmid pUA66_pcspA-ccdB_pLacUV5-ccdA was constructed amplifying the backbone and cspA region using cspA from pUA66_PcspA-GFP No Linker with T1+T2 and T5+T6 primers, respectively (Supplementary Table S2). Genes *ccdB* and *ccdA* were firstly amplified from the F-plasmid of the *E. coli* ER2738 strain with primers P1+P2 to clone the whole transcriptional unit in the repository vector pSB1C3. The *ccdA* was amplified with T3+T4, while *ccdB* gene was amplified as two separate pieces to remove an internal, undesired, Bsa-I site with T7+T8 and T9+T10 primers.

To test if the XylS2/P_m_ expression system variants were sensitive to salicylic acid derivatives, plasmids pSEVAb238_XylS2(R45T)/P_m_-sfGFP and pSEVAb238_XylS2(A111V)/P_m_-sfGFP were used. To construct them, the pSEVAb238_XylS/P_m_-sfGFP backbone was amplified with the I1 and I2 primers from an in-house plasmid template. The *xylS* gene was amplified in two fragments to introduce the point mutations necessary to create the *xylS2*(R45T) and *xylS2*(A111V) variants. Two Golden Gate assembly reactions with three fragments (backbone, first half of *xylS* and second half of *xylS*, containing the point mutation) were then performed. The sequence of the insert was verified using primers U5 and U6.

The plasmid pSEVAb23_NahR/P_sal_-sfGFP was built for the same objective, which was carried out as follows. First, the sequence of the ASA-inducible NahR/P_sal_ system was obtained from the pSB1C3_NahR/P_sal_ plasmid, acquired from the iGEM registry (BBa_J61051) and verified using the U1 and U2 primers. Then, it was amplified with the primers I11 and I12. Next, the sequence of the destination backbone, pSEVAb23_XylS/P_m_-sfGFP, was amplified with the I9 and I10 primers from an in-house plasmid template. Finally, assembly of the two pieces was performed using Golden Gate. The verification of the sequence was carried out through sequencing using the primers U5 and U6.

The plasmid used to express the type I-C CRISPR-Cas system, pCas3cRh originates from the work by Csörgő et al. (2020). It has a kanamycin-resistance gene and contains the CRISPR array and the *cas3, cas5, cas8* and *cas7* genes under the RhaSR/P_rhaBAD_ rhamnose-inducible expression system. The CRISPR array is designed in such a way that the spacer sequence can be easily interchangeable by restriction-ligation with the type II restriction enzyme BsaI. The spacer sequence between the repeats in the original plasmid is random and does not target the *E. coli* DH5α or EcN genome. Moreover, the sequence of the second repetitive sequence is slightly modified to avoid spacer flip-out by recombination (Csörgő et al., 2020).

### XylS engineering

Two mutations (R45T and A111V) were implemented in the *xylS* gene to create the variants *xylS2*(R45T) and *xylS2*(A111V), with the objective of testing their response to aspirin. To introduce the point mutations, one forward and one reverse flanking primers were designed at the *xylS* flanks (I3 and I4, respectively) to amplify the gene from the in-house pSEVAb238_XylS/P_m_-GFP plasmid. Next, two internal primers were designed for each point mutation. The internal primer in the forward direction was located at the point of the gene to be mutated and contained the nucleotide change (primer I5 for R45T and primer I7 for A111V), while the reverse primer overlapped a few nucleotides with the forward primer but was not positioned on the nucleotide to be mutated (primer I6 for R45T and primer I8 for A111V). All primers contained the necessary overhangs for further Golden Gate assembly. Two PCR reactions for each variant were then performed using NEB Q5 High-Fidelity DNA Polymerase. For the *xylS2*(R45T), the primer pairs I3 + I6 and I4 + I5 were used. For *xylS2*(A111V), the primer pairs I3 + I8 and I4 + I7 were employed.

### Spacer design and assembly

One of the REP sequences identified in *E. coli* K12 by Ton-Hoang et al. (2012) was found repeated twenty times in the genome of EcN, and in five cases it was adjacent to the 5′-TTC-3′ PAM sequence, allowing us to design a spacer to target these regions. The spacer was designed as a pair of complementary sense and antisense oligos (O1 and O2), adding overhangs to the sequence to allow them to bind to pCas3cRh after digestion with BsaI. Therefore, the forward oligo had the sequence 5′-gaa​acT​TGC​CGG​ATG​CGG​CGT​AAA​CGC​CTT​ATC​CGG​CCT​g-3′, and the reverse oligo had the sequence 5′-gcg​acA​GGC​CGG​ATA​AGG​CGT​TTA​CGC​CGC​ATC​CGG​CAA​g-3’. The assembly of the crRNA cassette was performed by annealing the forward and reverse oligos by temperature ramp down. The reactions were prepared by adding 1 µL of each oligo and 2.5 µL of NaCl to a final volume of 50 μL, and incubated at 95 °C for 5 min, after which the thermocycler was switched off and the samples left for 2 h in the machine, so that the temperature would slowly drop to room temperature. After the annealing reaction, the oligos were diluted 100-fold. pCas3cRh was digested with the restriction enzyme BsaI-HFv2 for 15 min at 37°C, followed by heat inactivation of the enzyme for 20 min at 80°C. Next, the digested plasmid samples were cleaned using the Nucleospin Gel and PCR clean-up (Machery-Nagel) kit and concentrated by eluting in 11 μL of miliQ water. The ligation of the spacer in pCas3cRh was performed in a final volume of 20 μL, where 150 ng of digested pCas3cRh, 5 µL of the annealed spacer, 5 µL of T4 DNA ligase buffer 10X and 1 µL of T4 DNA ligase were added. The ligation mix was incubated at room temperature for 10 min after which the T4 DNA ligase enzyme was inactivated at 65 °C for another 10 min. Finally, 5 μL of the ligation mix was transformed by heat-shock into chemically competent *E. coli* DH5α.

### Growth assay

Cell death assays were performed in a plate reader to test the killing efficiency of the CRISPR-Cas mechanism and to find out the optimal inducer concentration for its expression. Precultures were prepared by inoculating individual colonies of the strains to be tested in 10 mL of LB medium with the necessary antibiotics and grown overnight at 37°C and 250 rpm. The following day, the OD_600_ of each preculture was measured, and the cells were washed three times in M9 medium to ensure they were free of LB medium traces. Then, the washed cultures were inoculated with an OD_600_ of 0.1 in the different testing media. Next, 200 μL of each sample were pipetted into a 96-well plate, and 50 μL of mineral oil were carefully added on top to allow air exchange while avoiding contamination and noise in the measurements caused by condensation. Cell cultures were grown in 96-well plates in a BioTek Synergy Mx microplate reader machine for 24 h at 37 °C with double orbital continuous agitation, and OD_600_ measurements were taken every 5 min.

### Fluorescence assay

To test the response of the pcspA system and the chimeric receptor Dismed2:EnvZ, as well as the activity of the different inducible expression systems, fluorescence assays were performed. Overnight cultures were washed with M9 media twice before resuspension in M9 supplemented with 50 mM glucose and appropriate antibiotics, when needed. Starting OD_600_ was normalized to 0.3 for the inoculum of the plate reader experiment. Experiments were performed in the BioTek Synergy Mx microplate reader machine for 24 h at a temperature of 37°C with continuous shaking and measurements of OD_600_ and green fluorescence (excitation λ: 467 nm, emission λ: 508 nm, gain: 50) every 5 min.

To test the pUA66_PcspA-GFP No Linker system in EcN, three experiments were performed with different incubation temperatures: 37°C, 30°C, 26°C. In the experiments performed at 30°C and, 26°C, controls pre-cultured at these temperatures were also added, respectively.

The functioning of the chimeric receptor Disemed2:EnvZ was analysed with the EcN strain harbouring both pSEVAb22_Dismed2:EnvZ and pSB1C3_pOmpC-GFP alongside with the controls EcN equipped with pSB1C3_pOmpC-GFP and pSEVAb22_Dismed2:EnvZ and the wild type (WT) EcN. The test and the two controls were pre-cultured overnight in biological replicates in LB medium only. For the microplate reader under following increasing concentrations of mucin: no mucin, 0.1% (v/v), 1% (v/v), 5% (v/v) and 10% (v/v).

### Temperature-sensitive survival assay

The EcN strain harbouring the temperature sensitive promoter pcspA controlling the ccdA-ccdB toxin-antitoxin system was tested by means of a temperature survival assay. For this, chemically competent EcN cells were transformed with pUA66_pcspA-ccdB_pLacUV5-ccdA. After transformation, cells were subjected to a preliminary test by plating them directly onto different plates which were subsequently incubated at different temperatures: 37°C, 30°C, 25°C, 20°C. CFU grown at 37°C were later re-streaked onto plates subsequently incubated at lower temperatures to test for escapers. Ultimately, CFU which did not show any growth at lower temperatures were cultured overnight in liquid LB and antibiotics, and the day after were again plated to be incubated at the same four temperatures indicated above. Kill switch survival was calculated after 48 h as the ratio between CFU growing upon non-permissive conditions (30°C, 25°C, or 20°C) and CFU growing under permissive conditions (37°C), and expressed in percentage.

### Statistical analysis

Retrieved data from the fluorescence assays were analysed by calculating the relative fluorescence (Fluorescence/OD_600_) and by averaging the technical replicates of each biological replicate. Averages and standard deviations were then calculated of the biological replicates (*n* = 3, unless otherwise stated) of each condition/sample. Statistical analyses were done by one-way Anova test or *t*-test (as stated in the text). Calculations were performed with Microsoft Excel and data visualisation was done with Microsoft Excel and Matlab version R2021b.

## Results

### A mucin auxotrophy restricts colourectal to the colon environment

**FIGURE 2:**
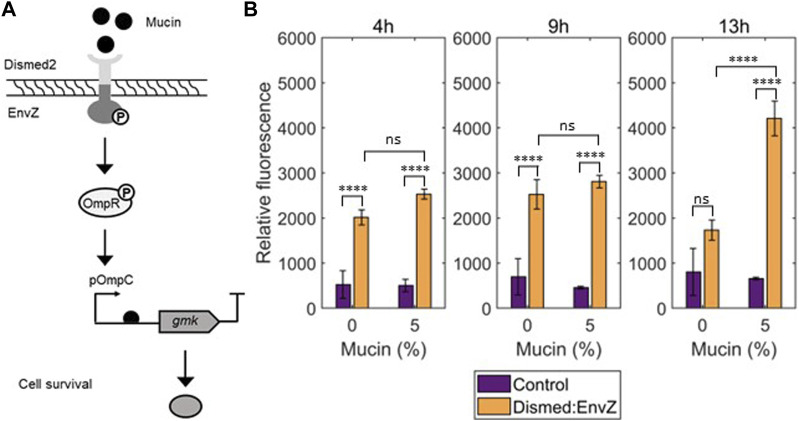
**(A)** The chimeric receptor, built as a two-component system (TCS), is formed by the extracellular Dismed2 domain of the RetS protein from *Pseudomonas aeruginosa* and the intracellular HisKA and HATPase intracellular domains of the EnvZ part of the EnvZ-OmpR TCS. In this system mucin is the activator of the chimeric TCS which is linked to the expression of the essential EcN gene *gmk*. **(B)** Detailed point results after 4 h, 9 h, and 13 h of incubation of fluorescence assay to test the *E. coli* JW3367-3 ∆envZ strain with pSEVAb22_ Dismed2:EnvZ and pSB1C3_pOmpC-GFP with different mucin concentrations: no mucin; 0.1%; 1%; 5% and 10% mucin. Control = the *E. coli* JW3367-3 ∆envZ carrying pSB1C3 pOmpC-GFP. Technical duplicates as well as biological triplicates were included for all the conditions (Mean ± s.d., *n* = 3 biological). Statistical analyses were performed with one-way ANOVA test where ****p < 0.0001; ns *p* > 0.05.

To create the mucin auxotrophy, our system made use of a mucin-sensitive Dismed2 sensor and the well-studied EnvZ/OmpR two-component system (TCS) (Levskaya et al., 2005; Ganesh et al., 2017), creating the chimeric receptor Dismed2:EnvZ (Figure 2A). The Dismed2 domain from *P. aeruginosa*’s RetS receptor, which had been previously shown to respond to mucin presence (Ryan Kaler et al., 2021; Wang et al., 2021), was fused with the intracellular HisKA and HATPase intracellular domains from *E. coli*’s EnvZ. In essence, we hypothesized that upon sensing of mucin, Dismed2 would activate the transmitter domain of EnvZ. When activated, EnvZ phosphorylates the transcription factor OmpR, which subsequently activates the OmpC promoter (Kenney and Anand, 2020). As depicted in Figure 2A, in our strategy OmpC would regulate the transcription of *gmk*, an essential gene for cell survival due to its role in nucleotide metabolism (Baba et al., 2006; Gallagher et al., 2015). When mucin is present (permissive conditions), the essential gene would be expressed allowing cell survival. By contrast, in the absence of mucin (non-permissive conditions), *gmk* would not be expressed, rendering the cell non-viable.

As a proof-of-principle, we tested the Dismed2:EnvZ chimeric receptor in the *E. coli* JW3367-3 ∆*envZ* strain lacking native EnvZ (Baba et al., 2006). In place of the essential gene *gmk*, we used a *gfp* fluorescent reporter as the system read-out. We assessed the system with increasing concentrations of mucin (0%, 0.1%, 1%, 5%, and 10%). This range was chosen assuming that the majority of proteins in the mucus is mucin (Melhem et al., 2021). In the assay, the chimeric receptor and *gfp* were expressed in plasmids pSEVAb22_Dismed2:EnvZ and pSB1C3_pOmpC-GFP, respectively. As a control, we used an *E. coli* JW3367-3 ∆*envZ* strain equipped with pSB1C3_pOmpC-GFP and an empty pSEVAb22 vector to observe background fluorescence and analyse to what extent the chimeric receptor responds to mucin. Three major effects can be observed according to the results depicted in Figure 2B (and Supplementary Figure S1). First, basal levels of fluorescence are shown in the control compared to the test strain, highlighting that the system gets partially activated even in the absence of EnvZ. Second, more fluorescence was observed in the test strain when compared to the control regardless of mucin availability or time. This reveals the leakiness of our system such that the system functions even in absence of the required input. Consequently, cells would survive even in the absence of mucin. Third, we see that the positive effects of mucin on system function alter with time, whereby cells would require a long presence of mucin for population growth. Overall, this suggests that the system does function as desired, but may result in undesired viable cells under non-permissive conditions.

### Temperature-sensitive kill switch renders colourectal non-viable at temperatures lower than 37°C

**FIGURE 3:**
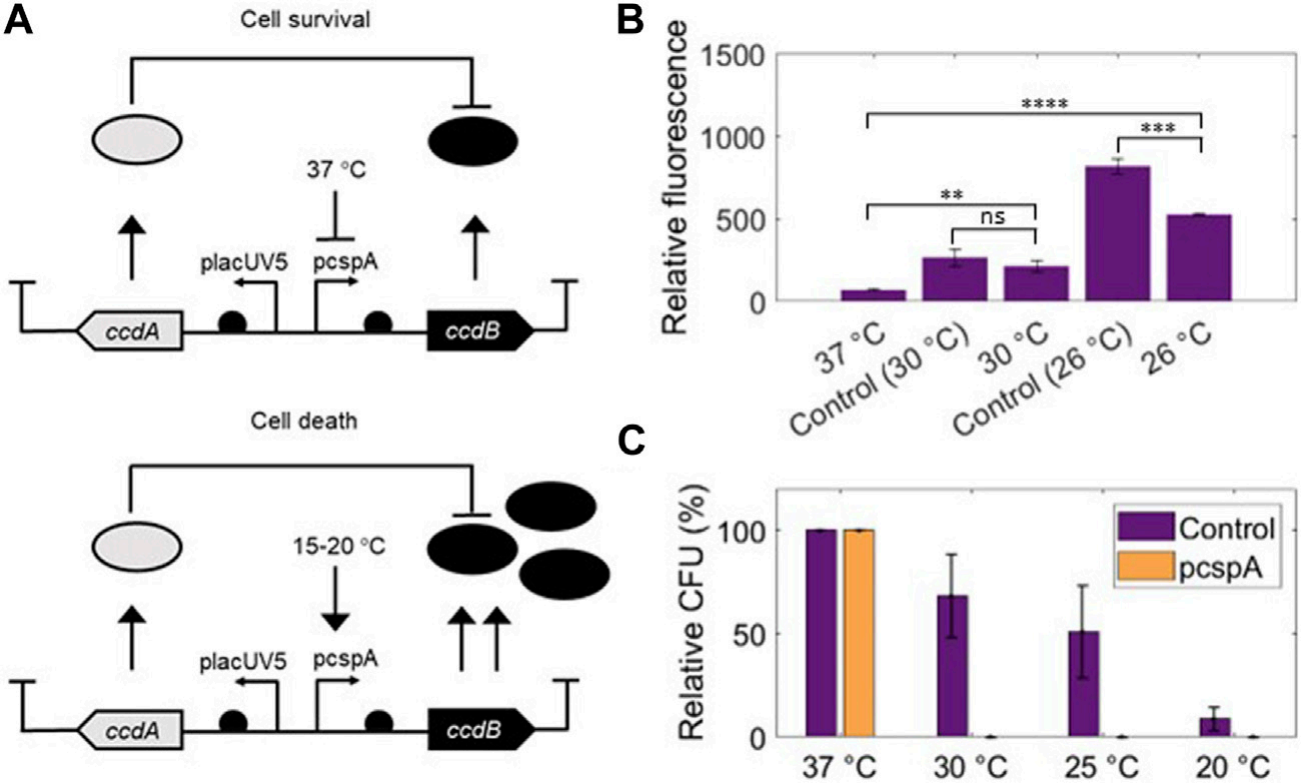
**(A)** The promoter pcspA is located upstream of the toxin (ccdB) while the antitoxin (ccdA) is placed under the control of the constitutive LacUV5 promoter. When temperature decreases, the expression of the toxin increases, leading to the microorganism’s death. Figure based on the design from Riglar and Silver (2018). **(B)** Detailed point results after 6 h of incubation of the fluorescence assay of EcN pUA66_PcspA-GFP No Linker at different temperatures. Control 30°C = pre-culture at 30°C, control 26°C = pre-culture at 26°C. Technical triplicates as well as biological triplicates were included for all the conditions (Mean ± s.d., *n* = 3 biological). Statistical analyses were done with one-way ANOVA test where ****p < 0.0001; ***p < 0.00017; ns *p* > 0.05. **(C)** Temperature sensitive survival assay of the EcN strain carrying pUA66_pcspA-ccdB_pLacUV5-ccdB performed at different temperatures: 37°C, 30°C, 25°C, 20°C. Control = EcN strain harbouring pUA66 No Linker plasmid. Biological replicates for all the conditions (Mean ± s.d., *n* = 3 biological). Relative CFU was calculated both for the pcspA and the control strains as the ratio between CFU growing upon non-permissive condition (30°C, 25°C and 20°C) and the total CFU growing on plates in permissive condition (37°C) and expressed in percentage.

The temperature-sensitive regulatory region of cold shock protein A (CspA) has been previously harnessed for a temperature-sensitive genetic circuit in *E. coli* Dh10β (Stirling et al., 2017). The constitutive promoter of CspA (pcspA) has a high rate of transcription but contains a long 5′ untranslated region (UTR) that, at 37°C, forms an unstable secondary structure resulting in degradation of transcribed mRNA (Mitta et al., 1997). In contrast, at lower temperatures, pcspA has a more stable conformation, which allows the translation of downstream genes (Giuliodori et al., 2010). In this version of the kill-switch, the toxin/antitoxin ccdAB pair is used to control cell death (Figure 3A, Stirling et al., 2017). This system is naturally encompassed within the F-plasmid of *E. coli,* which encodes the *ccdA* and the *ccdB* genes whose products function as the system antidote and poison, respectively (Bahassi et al., 1999). To exert its killing mechanism, ccdB binds the GyrA subunit of the DNA gyrase protein, an essential bacterial enzyme (Reece and Maxwell, 1991), and prevents its proper functioning during DNA replication. This negative impact leads to DNA breakage, resulting in cell death (Bernard et al., 1993). The toxin/antitoxin system chosen to construct the temperature kill switch does not interfere with mammalian cells, such as those found in the colon, as it specifically targets the DNA gyrase of Enterobacteriaceae (Wu et al., 2020). Moreover, the genetic circuit functions in such a way that leaky expression of the pcspA-driven toxin under permissive conditions would be counteracted by the antitoxin placed under the control of the constitutive promoter LacUV5 (Riglar and Silver, 2018).

The pcspA system was tested at three temperatures (37°C, 30°C and 26°C) in EcN. The warmest temperature, 37°C, was chosen as the permissive condition since it is both optimal for EcN growth and also the human body temperature. The cooler temperatures were chosen to test the temperature-sensitivity of the system. We were able to verify the published system’s functionality using a fluorescence reporter in place of the toxin (Stirling et al., 2017). All pre-cultures were grown overnight in LB at 37°C, which allowed us to test the response of the genetic circuit to a switch of temperature from 37°C (permissive conditions) to lower temperatures, 30°C and 26°C (non-permissive conditions). As a control, we also tested the system after 30°C and 26°C pre-cultures. Samples exposed to 30°C and 26°C showed levels of fluorescence significantly higher than those of the 37°C sample after 6 h of incubation regardless of pre-culture conditions (Figure 3B; Supplementary Figure S2). Qualitatively, the results are in line with reported literature (Stirling et al., 2017) since the system functions optimally at higher temperatures compared to lower ones, showing that, in principle, our temperature-sensitive system could effectively be used in EcN.

To generate the temperature-sensitive kill switch (pUA66_pcspA-ccdB_pLacUV5-ccdB) we substituted the fluorescence reporter with the toxin/antitoxin system. To assess its killing capabilities, a cell survival assay was performed with EcN carrying the temperature-sensitive kill switch. This system was evaluated under four different temperatures: 37°C (permissive conditions), 30°C, 25°C, and 20°C (non-permissive conditions). The same protocol was followed for the control EcN strain (carrying the plasmid pUA66_PcspA-GFP No Linker). After 48 h of incubation, CFU counting was performed to calculate the survival rate of cell colonies. On one hand, controls showed a typical decrease in CFU with decrease of temperature due to slower growth (Kumar and Libchaber, 2013). On the other hand, when compared to the control, the EcN strain showed a dramatic CFU decrease at temperatures lower than 37°C, since no colonies were observed on the plates incubated at 30°C, 25°C and 20°C (Figure 3C; Supplementary Table S4). From these results, we conclude that the temperature-sensitive kill switch has 100% efficacy at temperatures below 37°C and would efficiently kill Colourectal in environments outside of the body.

### Type I-C CRISPR-cas-based rhamnose-inducible kill switch produces strong genotoxic response in EcN

To develop a test terminator/emergency kill switch, we leveraged the lethal power of type I-C CRISPR-Cas. First, in the search for a suitable activation system for our CRISPR-based kill switch in EcN, we tested multiple inducible expression systems: benzoate-inducible XylS/P_m_, L-arabinose-inducible AraC/P_araBAD_, L-rhamnose-inducible RhaSR/P_rhaBAD_, and aspirin-inducible XylS2/P_m_ and NahR/P_sal_ systems. We sought to identify an inducible expression system that showed a high dynamic range, defined by the ability of achieving high levels of gene expression when induced, but minimal basal (“leaky”) expression in the absence of inducer.

Fluorescence assays were performed in EcN strains expressing *sfgfp* under the control of the aforementioned inducible expression systems (Supplementary Figure S3). No fluorescence was detected in aspirin-inducible systems in the presence of inducer when compared to the negative controls. In addition, the AraC/P_araBAD_ system showed a low dynamic range in EcN, since high expression of *sfgfp* was detected in the absence of inducer. Therefore, only XylS/P_m_ and RhaSR/P_rhaBAD_ showed the desired characteristics. As the L-rhamnose-inducible expression system is native to *E. coli*, we continued our system design with RhaSR/P_rhaBAD_.

**FIGURE 4:**
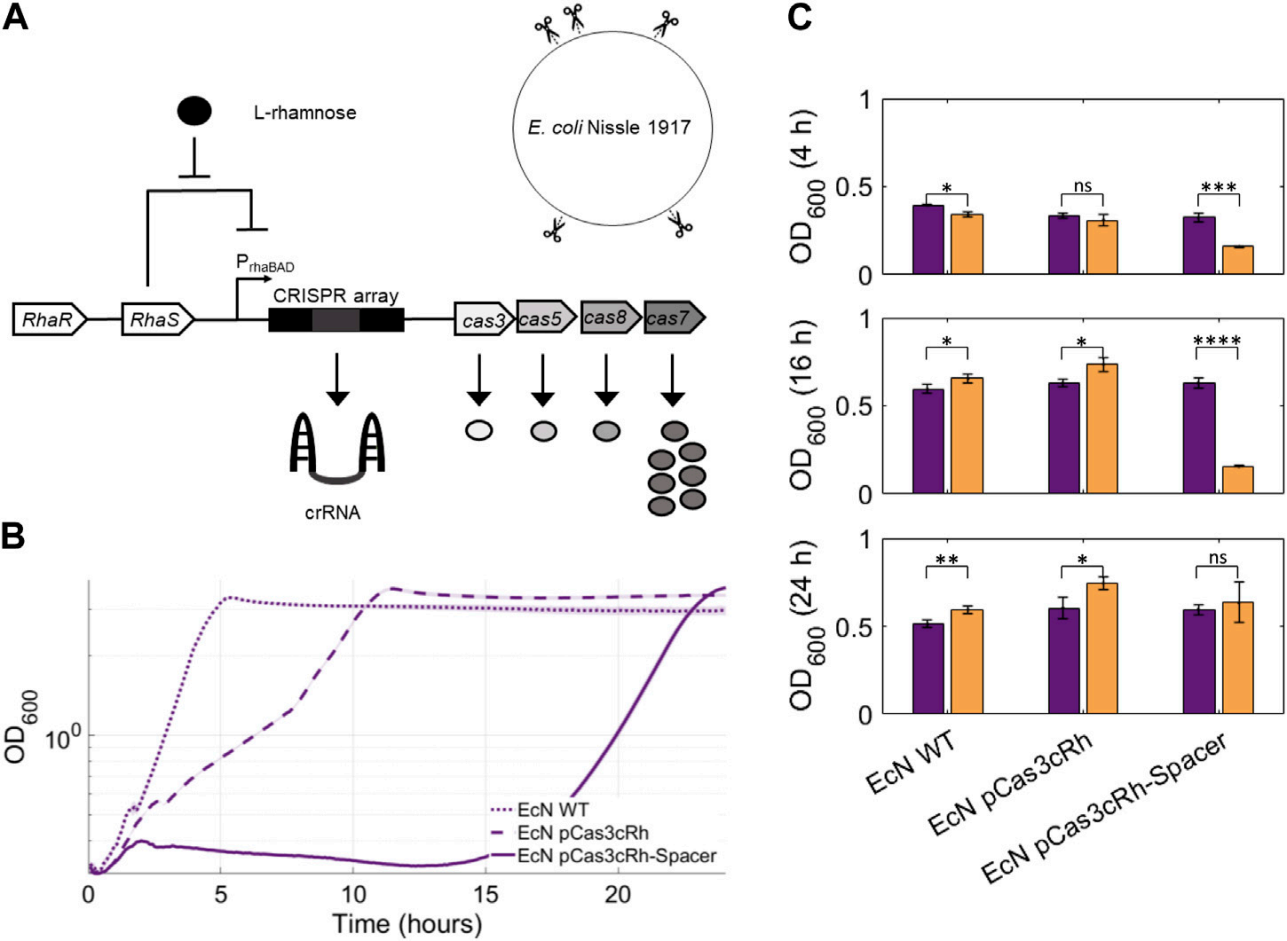
**(A)** Schematic of the CRISPR-based rhamnose-inducible kill switch circuit. CRISPR cassette expression was controlled by the RhaSR/PrhaBAD inducible expression system. Type I-C CRISPR-Cas effector module (Cascade) is comprised of seven Cas7, one Cas8, one Cas5 and one crRNA. Cascade scans the genome for a sequence complementary to the spacer sequence present in the crRNA. If the target sequence is found adjacent to the 5′-TTC-3′ PAM sequence, Cas3 is recruited and cleaves it. We designed a spacer sequence targeting five loci in the genome of EcN, which we expected to result in high genotoxicity. **(B)** Results of the CRISPR-based kill switch cell death assay. The growth curves of EcN WT, EcN pCas3cRh and EcN pCas3cRh-Spacer when 5 mM of rhamnose were added to the medium are compared. The optical density (OD600) of each sample over 24 h is shown. All samples were grown in M9 medium with 50 mM of glucose and 5 mM of rhamnose. Technical triplicates were included in all conditions (mean ± s.d., *n* = 3 technical). **(C)** Comparison of EcN WT, EcN pCas3cRh and EcN pCas3cRh-Spacer OD600 values between uninduced and induced conditions at three different time points. Samples with no inducer (purple bars) were grown in M9 medium with 50 mM of glucose. Induced samples (orange bars) were grown in M9 medium with 50 mM of glucose and 5 mM of rhamnose. Both technical and biological triplicates were included in all cases (mean ± s.d., *n* = 3 biological). Statistical analyses were carried out using a two-tailed *t*-test where ****p ≤ 0.0001, ***p ≤ 0.001, **p ≤ 0.01, *p ≤ 0.0.05, n.s. *p* > 0.05.

To initiate cell death in the presence of our inducer, we used the type I-C CRISPR-Cas system due to its compact size and the helicase-nuclease activity of Cas3. Cas3 continuously degrades target DNA creating large genomic deletions that we presumed would be highly genotoxic (Csörgő et al., 2020). We made genomic interference L-rhamnose-inducible by placing both the CRISPR array and the four *cas* genes (*cas3*, *cas5*, *cas8* and *cas7*) under the control of the P_rhaBAD_ expression system (Figure 4A). Next, for our crRNA, a spacer sequence was sought that would target genomic regions and provide our system with a high killing efficiency. We hypothesised that one with multiple target points in the genome of EcN would result in increased genotoxicity compared to targeting a single position (Castanon et al., 2020). Hence, a repetitive extragenic palindromic (REP) sequence found twenty times in the genome of EcN, in five cases adjacent to the type I-C CRISPR-Cas PAM sequence (5′-TTC-3′), was chosen as the target genomic location.

To assess the killing efficiency of crRNA targeting REP sequences, growth experiments were performed in a plate reader in which absorbance (OD_600_) measurements–a readout for cell population size–were compared between permissive (- rhamnose) and non-permissive (+rhamnose) conditions over time. Additionally, controls including EcN WT and EcN pCas3cRh containing a non-targeting spacer were tested in the same conditions to assess if the addition of rhamnose gave rise to changes in the growth pattern of EcN and to observe the effect of CRISPR cassette expression in the cells when not targeting the genome, respectively.

First, we investigated kill switch activity by comparing the growth curves between EcN WT, EcN pCas3cRh and EcN pCas3cRh_Spacer strains upon addition of 5 mM L-rhamnose (Figure 4B). These results show a large genotoxic effect of the kill switch, delaying the growth of EcN pCas3cRh-Spacer strain more than 15 h after induction. The growth curve of the EcN strain with the pCas3cRh plasmid, which contained a non-targeting spacer and therefore could not cleave the EcN genome, showed slower growth (and longer lag time duration) than the EcN WT strain. It was hypothesised that slower growth occurred because the expression of the CRISPR cassette was generating metabolic burden in the cells, as the cellular machinery that was being used in the transcription and translation of the Cas proteins was not being used for other cellular processes. This is in line with previous studies that have shown that Cas protein expression consumes a significant amount of cellular energy and resources, which can reduce the overall growth rate and viability of the bacteria (Zaayman and Wheatley, 2022). The inducer concentration selected for the activation of the kill switch, 5 mM L-rhamnose, was deemed optimal based on the results of a previous cell death assay where we compared the growth curves of EcN pCas3cRh and EcN pCas3cRh_Spacer at increasing concentrations of L-rhamnose, ranging from 100 μM to 10 mM (Supplementary Figure S4).

Subsequently, a new experiment was performed on the same strains, but this time only comparing their behaviour with and without 5 mM L-rhamnose. Technical and biological triplicates of each sample were included, and a *t*-test was performed between samples with (orange bars) and without inducer (purple bars) at different time points (t = 4, 16 and 24 h) to verify if the difference in their growth was statistically significant (Figure 4C). After 4 h, a significant reduction in the induced EcN pCas3cRh_Spacer cell growth compared to the strain without inducer was found, meaning that the kill switch was activated and was effectively killing the cells. The results were similar at 16 h, where EcN pCas3cRh_Spacer showed a very significant reduction in population growth in the presence of inducer compared to the uninduced culture. However, at 16 h, EcN WT and EcN pCas3cRh showed improved growth in the presence of L-rhamnose, which could be caused by the cells starting to consume rhamnose in addition to glucose. Finally, 24 h after the start of the experiment EcN pCas3cRh_Spacer regained growth, and the difference with the non-induced culture was not significant anymore. We hypothesise that this is probably due to the presence of escaper mutants that repopulated the culture. The other two strains showed significantly higher OD_600_ values in the presence of L-rhamnose in comparison to its absence.

## Discussion

Embedding biocontainment strategies within a living diagnostic or biotherapeutic is a requirement for safe application use. To begin with, we chose a safe chassis, EcN, to build Colourectal. Furthermore, we built a multi-layered approach to ensure safety for the user and the environment. To ensure safety for the user, we built a mucin auxotropy, while for environmental safety, we used a temperature-sensitive genetic circuit. These two systems would allow the survival of our tool only under permissive conditions: the colon environment and body temperature. Since we were unsure that possible adverse reactions may occur or how long our tool would stay in the colon, we also included an emergency kill switch to allow the removal of Colourectal from the body at any given time.

We constructed the first layer of our biocontainment strategy with the synthetic chimeric receptor Dismed2:EnvZ, which has the capacity to activate the EnvZ/OmpC TCS. We showed that, after 13 h of induction by mucin, the system becomes activated (Figure 2B). However, basal reporter gene expression levels were also observed in the absence of mucin. As the EnvZ/OmpC TCS is naturally present in EcN, this could be a result of crosstalk between native and synthetic systems, which might cause the activation of the system when permissive conditions are not met (Morey et al., 2012). Even though the use of a Δ*envZ* strain should help reduce the crosstalk, the system is still activated, possibly due to the activation of the transcription factor OmpR by other mechanisms (Matsubara et al., 2000). A similar pattern was also reported in the work of Ganesh et al. (2017), that used a chimeric receptor constructed with EnvZ/OmpR to sense methanol. Activation of biocontainment systems in the absence of an input would limit the potential of this strategy that requires tight on/off switching. Specifically, the basal fluorescence levels of the OmpC promoter observed in the absence of mucin (Figure 2B) could potentially have a detrimental effect on our auxotrophy strategy if very low expression levels of the controlled essential gene, *gmk*, sustained cell survival. To test this in the future, it would be important to test inducible *gmk* expression within a *Δgmk* strain lacking any native *gmk* production. To overcome this potential obstacle, one possibility is to utilise a different TCS in our system that shows reduced basal reporter gene expression in the negative controls. As TCSs are ubiquitous systems among microorganisms (Lazar and Tabor, 2021), a non-native system could be used. Ultimately, the choice should be made based on: (i) the performance required from the TCS and (ii) the absence of the chosen TCS in the strain of interest.

To add to this, growth curves (Supplementary Figure S1) showed that the cell population of EcN strains grown in the presence of mucin show faster exponential growth. This is likely due to adaptation of EcN to use galactose as a carbon source for energy, which is one of the main mucin components (Tailford et al., 2015; Crook et al., 2019). The fact that EcN can use mucin as a growth substrate could interfere with the envisioned auxotrophy. This highlights the limitation in the use of a native molecule as input for auxotrophic systems that require tight, coordinated control (Rottinghaus et al., 2022). An interesting alternative to the use of mucin would be to make EcN dependent on quorum sensing molecules or on metabolites produced by the other bacteria of the gut microbiota. Although difficult, due to the lack of consensus on the microbiota composition (Redondo-Useros et al., 2020; Cani et al., 2021), and inter-individual variability of these microbiota (Healey et al., 2017), previous research has explored the use of homoserine lactones (HSLs) as signalling molecules for cell-to-cell communication (Tekel et al., 2019; Lazar and Tabor, 2021).

To avoid environmental escape, we used a temperature-sensitive genetic circuit (Stirling et al., 2017). We first tested the behaviour of the pcspA genetic circuit with a fluorescent reporter gene in EcN, showing that with decreasing temperatures, higher fluorescence levels were achieved. The result was in accordance with previous research performed in other *E. coli* strains (Stirling et al., 2017). We then tested the mechanism of the genetic circuit with the ccdA/ccdB toxin/antitoxin system and observed 100% killing efficiency (Figure 3C). In this case, our result is not completely in line with previous literature. High expression levels of the pcspA regulatory region were shown to be achieved around 15°C–20°C (Fang et al., 1999; Stirling et al., 2017), whereas in our case we saw that the killing mechanism was already active at 30°C, suggesting that the promoter region is more sensitive than previously shown. If the activation of the genetic circuit occurs already at 36°C, then the ccdB toxin would be released into the colon environment. This might be harmful for the colon microbiota (but not human cells), since the ccdB toxin targets the DNA gyrase of bacteria belonging to the Enterobacteriaceae family (Wu et al., 2020), which is one of the most abundant family of the phylum Proteobacteria in the human microbiome (King et al., 2019) Therefore, even though we proved the efficiency of our system, we propose that further experiments should be done to properly assess temperature ranges between 37°C and 30°C.

Finally, we developed an inducible type I-C CRISPR-Cas-based kill switch targeting five REP sequences in the genome of EcN as a strategy to remove Colourectal from the colon on demand. Type I-C CRISPR-Cas activity resulted in high genotoxicity in EcN when induced (Figure 4C), demonstrating that systems based on the action of Cas3 present a powerful tool in the construction of specific and highly genotoxic kill switches, as shown in previous studies (Gomaa et al., 2014; Caliando and Voigt, 2015). For the development of such a circuit, we first characterised multiple inducible expression systems in EcN to find the most suitable one. Although ASA initially appeared to be an adequate inducing molecule for this third genetic safeguard of Colourectal, we learned that there were certain practical limitations to the use of this molecule. Notably, ASA is widely used in small daily doses for the prevention of cardiovascular problems (McNeil et al., 2018). Consequently, activation of the kill switch would be undesirably triggered prematurely in people using ASA daily but also requiring colon treatment. This rendered ASA an unsuitable candidate for Colourectal.

Although this work has shown that the inducible RhaSR/P_rhaBAD_ expression system works satisfactorily for kill switch induction in EcN (*i.e*., it does not generate detectable leakiness, produces a strong, dynamic response, and is native to *E. coli*; Supplementary Figure S4E), we believed that it would not be the most appropriate system for the final design of Colourectal. Rhamnose is present in some foods, such as oranges, beans, cabbage, and carrots, and reaches the colon intact once ingested (Vogt et al., 2004). This could pose a problem as rhamnose consumed in the diet may be sufficient to activate the kill switch when not desired. On the other hand, some microorganisms present in the colon are capable of metabolising rhamnose (Byrne et al., 2018), so it becomes difficult to determine how much inducer would be available for the induction of the CRISPR-Cas mechanism in EcN once the “test terminator” has been ingested to eliminate the living diagnostic from the body.

Previous living therapeutics have made use of tetracycline- (Tristán-Manzano et al., 2020), arabinose- (Cubillos-Ruiz et al., 2021), aspirin- (Medina et al., 2011), or even light- (Liu et al., 2021) inducible expression systems. However, none of these inducers fulfils all the required characteristics for Colourectal. The optimal inducer must not be present in the diet, in the colonic environment, must be safe for human health and for the microbiome, and must have a high dynamic response in EcN. An expression system inducible by such a molecule has not yet been found, but it would represent a breakthrough for the regulation of genetic circuits in living therapeutics or diagnostics that perform their function in the digestive tract. A potential solution would be the engineering of a synthetic inducible expression system activated by a molecule optimal for these applications, however research in this area is limited (Liu et al., 2019).

A further issue that we observed was the growth of cell populations in undesirable situations. The emergence of cells capable of surviving in non-permissive conditions, known as escaper mutants, is a common occurrence for virtually all types of kill switch circuits (Rottinghaus et al., 2022). On the one hand, no escaper mutants were detected when we tested our temperature-sensitive toxin-antitoxin kill switch (Figure 3C). While this absence of escapers is a good sign, the lack of escapers also prevented us from pursuing further investigations into the robustness of the system. Testing the escape frequency and performing analysis of genetic stability would have allowed us to obtain a more complete overview of the reliability of the system. For instance, testing the kill switch in larger culture volumes could allow the isolation of mutants even if the escape frequency is low. On the other hand, in the case of our rhamnose-inducible circuit, culture growth recovered completely after 24 h (Figure 4), in line with what was found in previous kill switches using CRISPR-Cas systems (Caliando and Voigt, 2015; Stout et al., 2018; Rottinghaus et al., 2022). Cell cultures that arose after activation of the kill switch were not studied during the course of this work, but sequencing of these strains would provide insight into the most frequent mutations. Type I-C systems targeting the genome have previously shown that mutations in *cas3*, the removal of the spacer, and mutations in the target sequence can deactivate their killing mechanism (Csörgő et al., 2020). By reducing the mutation rate, new strategies can be developed to increase the stability of the circuit. For instance, introducing redundant CRISPR cassettes into the genome, inserting multiple copies of the transcription factor of the inducible expression system, or inactivating the genes responsible for the SOS defence mechanism have proven to be effective strategies in decreasing the emergence of escaper mutants in CRISPR-Cas based kill switches (Caliando and Voigt, 2015; Rottinghaus et al., 2022). In future steps, it would be interesting to combine these strategies to ensure that the kill switch remains stable during the diagnostic process and that no escaper mutants appear that prevent its complete elimination from the user’s body when desired. Overall, we have shown that the temperature-sensitive and the rhamnose-inducible kill switches work in EcN, but further assessment should be done before presenting Colourectal as a safe/functional tool.

To summarise, we have presented a multi-layered biosafety and biocontainment strategy for living diagnostic or therapeutic bacteria is essential to ensure safe and effective use in clinical settings. With the increasing interest in using living bacteria to diagnose or treat various diseases, it is crucial to address potential safety concerns associated with their use. A comprehensive approach that includes multiple layers of safeguards, can minimize the risks of unintended consequences, and enhance the safety and efficacy of these innovative therapies. The results of this work provide a good foundation for the development and implementation of robust biosafety and biocontainment strategies in future living diagnostic or therapeutic microorganisms.

## Data Availability

The original contributions presented in the study are included in the article/Supplementary Material, further inquiries can be directed to the corresponding authors.

## Supplementary material

The supplementary tables and figures for this article can be found in the “Data Sheet 1.XLSX” and “Image 1.PDF” files, respectively.
