# Supplementary material for: Multilayered safety framework for living diagnostics in the colon
Source: Front Syst Biol. 2023 Sep 22;3:1240040. doi: 10.3389/fsysb.2023.1240040 (PMC12342019; doi:10.3389/fsysb.2023.1240040)
Supplement: Supplementary file 1 [file Image1.PDF]

Supplementary Material

Multilayered safety framework for living diagnostics in the colon

Sonia Mecacci[1†], Lucía Torregrosa-Barragán[1†], Enrique Asin-Garcia[1,2*], Robert W. Smith[1*]

1 Laboratory of Systems and Synthetic Biology, Wageningen University & Research, Wageningen, The Netherlands

2 Bioprocess Engineering, Wageningen University & Research, Wageningen, The Netherlands

† These authors share first authorship

*** Correspondence:**

Corresponding Authors robert1.smith@wur.nl and enrique.asingarcia@wur.nl

1 Supplementary Data

Data is provided in the Excel file (Supplementary tables S1, S2, S3 and S4).

Supplementary Table S1: stains used in this study.

Supplementary Table S2: plasmids used in this study.

Supplementary Table S3: primers used in this study.

Supplementary Table S4: CFU counts as presented in Figure 3 of the main text.

2 Supplementary Figures and Tables

2.1 Supplementary Figures

Supplementary Figure 1

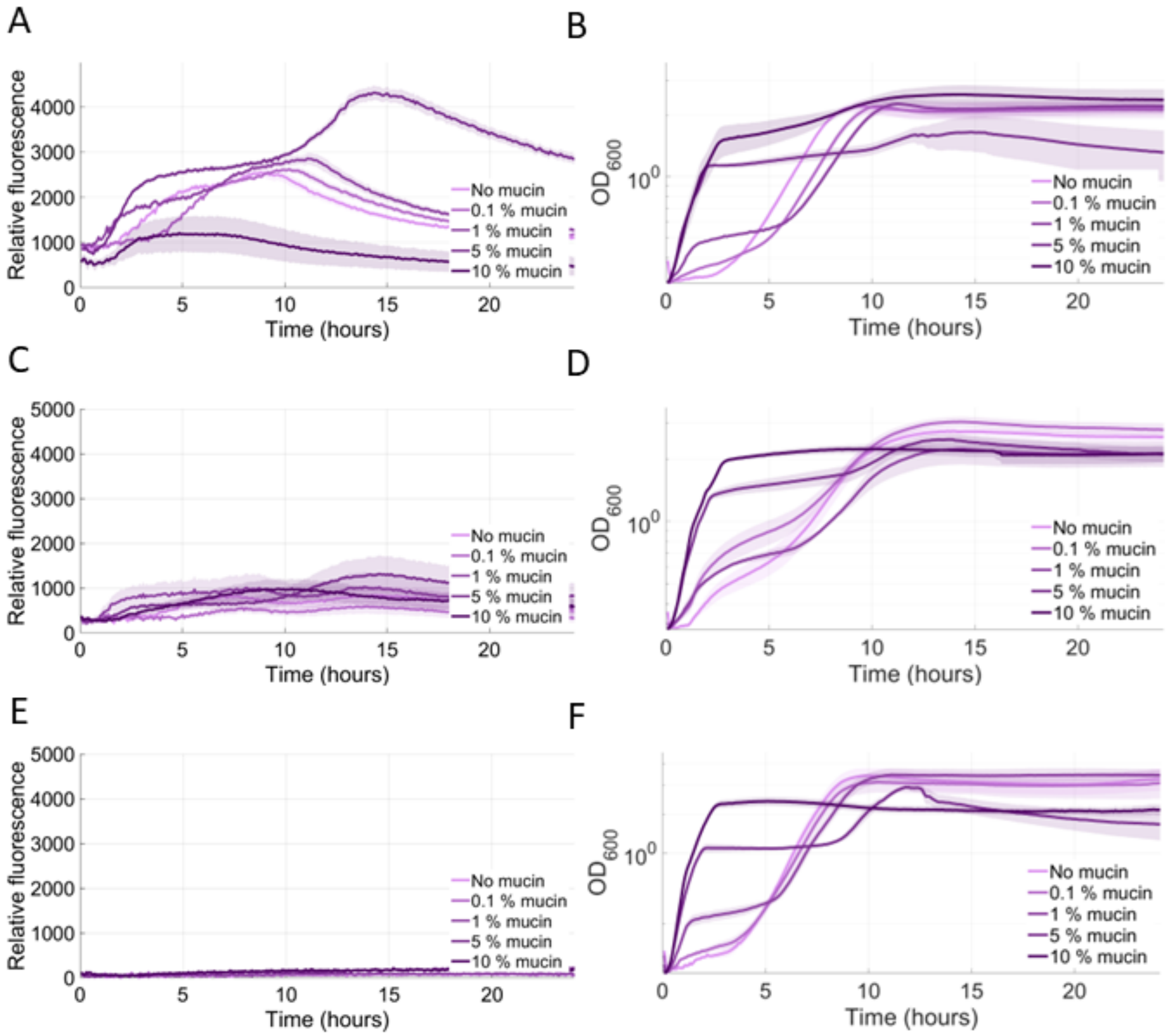

Supplementary Figure 1. Results of the fluorescence assay to test the chimeric receptor Dismed2:EnvZ with different mucin concentrations**.** Relative fluorescences levels of the *E. coli* JW3367-3 ΔenvZ strain with pSEVAb22 Dismed2:EnvZ **(A)** and the growth curves **(B)**, pSB1C3 pOmpC-GFP **(C)** and relative growth curves **(D)** and WT *E. coli* JW3367-3 ΔenvZ **(E)** and growth curves **(F)**. The strains were incubated with different mucin concentrations: no mucin; 0,1 %; 1 %; 5 % and 10 % mucin. Control 1 = E. coli JW3367-3 ΔenvZ strain with pSEVAb22 and pSB1C3 pOmpC-GFP. Control 2 = WT *E. coli* JW3367-3 ΔenvZ**.** Technical duplicates as well as biological triplicates were included for all the conditions (Mean ± s.d., n = 3 biological).

Supplementary Figure 2

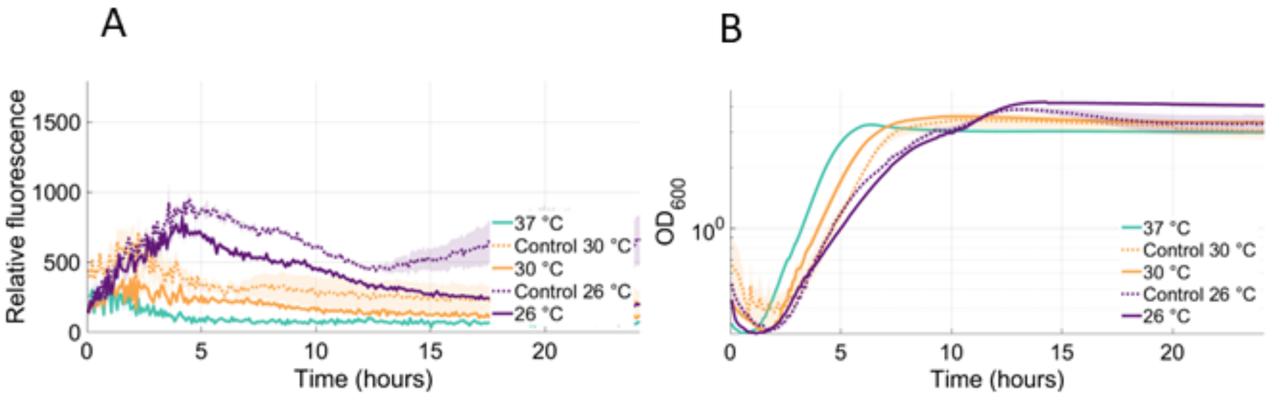

Supplementary Figure 2. Results of the fluorescence assay of EcN pUA66 PcspA GFP No Linker at different temperatures. The relative fluorescence levels of the different temperature conditions **(A)** and the growth curves **(B)** are plotted in a time series of 24 h. Control 30 °C = pre-culture at 30 °C, control 26 °C = pre-culture at 26 °C. Technical triplicates as well as biological triplicates were included for all the conditions (Mean ± s.d., n = 3 biological), with exception of one replicate from sample control 26 °C, which was eliminated due to contamination.

Supplementary Figure 3

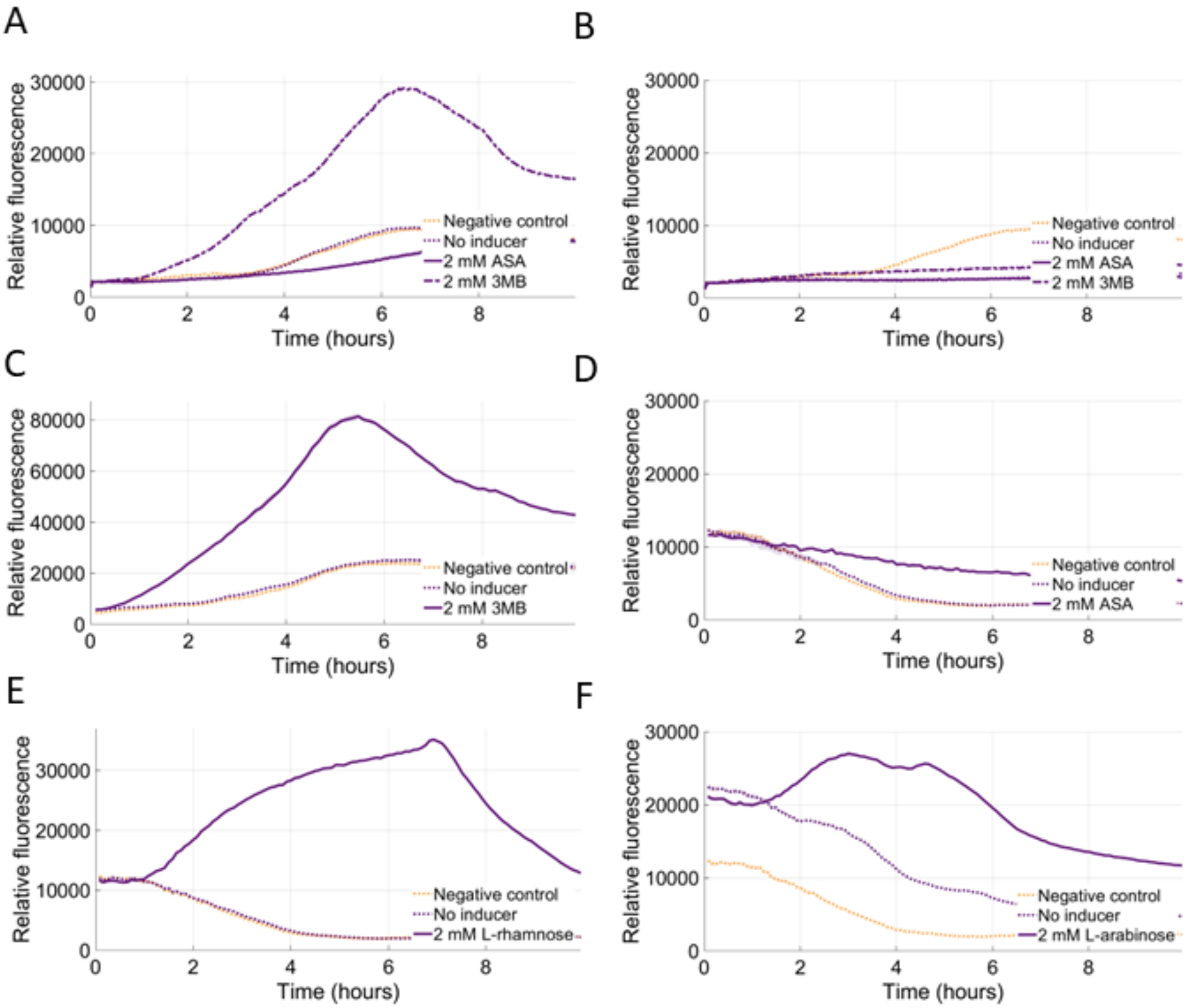

Supplementary Figure 3. Results of the fluorescence assay testing the different inducible expression systems in EcN. In each case, the relative fluorescence (absolute fluorescence/OD600) of each sample over a time period of 10 hours is presented. Negative controls contained the EcN WT strain, grown in M9 medium with 50 mM of glucose. Non-induced samples contained the EcN strains harbouring the plasmid with the respective inducible expression system, grown in M9 medium with 50 mM of glucose. Induced samples were grown in M9 medium with 50 mM of glucose and 2 mM of each inducer, to make the results comparable. Technical triplicates were included in all conditions (Mean +/- s.d., n = 3 technical). **(A)** XylS2(R45T)/P_m inducible expression system. **(B)** XylS2(A111V)/P_m inducible expression system. **(C)** XylS/P_m inducible expression system. **(D)** NahR/P_{sal} inducible expression system. **(E)** RhaSR/P_{rhaBAD} inducible expression system. **(F)** AraC/P_{araBAD} inducible expression system.

Supplementary Figure 4

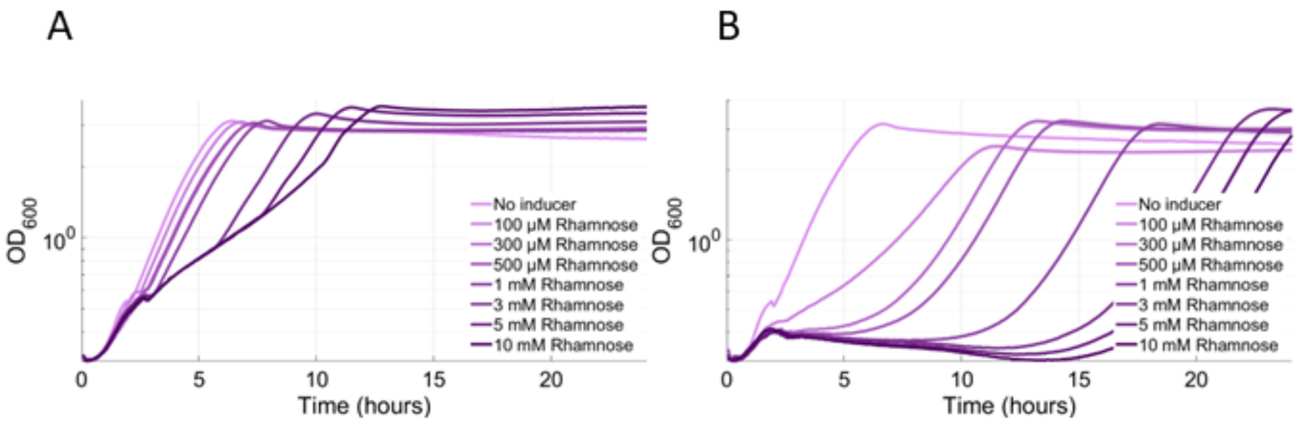

Supplementary Figure 4. Type I-C CRISPR-Cas cell death assay to find optimal inducer concentration. The optical density (OD_{600}) of each sample over 24 hours is shown. All strains were grown with no inducer (M9 with 50 mM of glucose) and in increasing rhamnose concentrations (100 µM, 300 µM, 500 µM, 1 mM, 3 mM, 5 mM, and 10 mM). Technical triplicates were included in all conditions (Mean +/- s.d., n = 3 technical). **(A)** Growth curves of the EcN pCas3cRh strain harbouring a non-targeting spacer. **(B)** Growth curves of the EcN pCas3cRh-Spacer strain. The spacer targets five REP sequences in the genome of EcN.
